# The Neural Substrates of Self-Evaluation of Mental Fatigue: A Magnetoencephalography Study

**DOI:** 10.1371/journal.pone.0095763

**Published:** 2014-04-21

**Authors:** Akira Ishii, Masaaki Tanaka, Yasuyoshi Watanabe

**Affiliations:** 1 Department of Physiology, Osaka City University Graduate School of Medicine, Osaka, Japan; 2 RIKEN, Center for Life Science Technologies, Kobe, Japan; Harvard Medical School/Massachusetts General Hospital, United States of America

## Abstract

There have been several studies of the neural mechanisms underlying sensation of fatigue. However, little is known about the neural mechanisms underlying self-evaluation of the level of fatigue. The aim of this study was to identify the neural substrates involved in self-evaluation of the level of mental fatigue. We used magnetoencephalography (MEG) with high temporal resolution on 14 healthy participants. During MEG recordings, participants were asked to evaluate their level of mental fatigue in time with execution cues (evaluation trials) or to do nothing in time with execution cues (control trials). The MEG data were analyzed with equivalent current dipole (ECD) and spatial filtering methods to localize the neural activity related to the evaluation of mental fatigue. The daily level of fatigue sensation was assessed using the Checklist Individual Strength questionnaire. In evaluation trials, ECDs were observed in the posterior cingulate cortex (PCC) in seven of 14 participants, with a mean latency of 366.0 ms. The proportion of the participants with ECDs in the PCC was higher in evaluation trials than in control trials (*P*<0.05, McNemar test). The extent of the decreased delta band power in the PCC (Brodmann’s area 31) 600–700 ms after the onset of the execution cue and that in the dorsolateral prefrontal cortex (DLPFC; Brodmann’s area 9) 800–900 ms after the onset of the execution cue were greater in the evaluation trials than in the control trials. The decrease in delta band power in the DLPFC was positively related to that in the PCC and to the daily level of fatigue sensation. These data suggest that the PCC and DLPFC are involved in the self-evaluation of mental fatigue.

## Introduction

Fatigue is a common problem. It has been reported that more than 20–30% of the general population complain of substantial fatigue in European countries and the United States [1]–[5]; in Japan, more than half of the adult population reports experiencing fatigue [6]. Fatigue can be defined as difficulty initiating or sustaining voluntary activity [7] and is accompanied by an unpleasant sensation. The sensation associated with fatigue plays an important role in biological alarm and urges us to take a rest to avoid disrupting homeostasis. However, over-estimation of the level of fatigue causes a severe fatigue sensation that can decrease physical and mental performance, resulting in further fatigue. In fact, an exaggerated fatigue sensation is thought to be involved in the pathophysiology of fatigue-related diseases such as chronic fatigue syndrome [8]. In contrast, under-estimation of the level of fatigue may cause overwork, which can be a cause of death in a working population, known as *karoshi* in Japan [9], [10]. Therefore, it is important to accurately evaluate the level of fatigue such that the intensity of the fatigue sensation matches the level of fatigue. Although several studies have investigated the brain regions related to fatigue sensation [11]–[14], little is known about the neural mechanisms underlying self-evaluation of fatigue level.

In our previous study we focused on the neural mechanisms related to self-evaluation of the level of physical fatigue [15]. We used magnetoencephalography (MEG) and performed equivalent current dipole (ECD) analysis to examine brain activity while participants were evaluating the level of physical fatigue in their right hand, and showed that the posterior cingulate cortex (PCC) was involved in the evaluation of physical fatigue [15]. It has been reported that the PCC is involved in self-monitoring and self-reflection [16]–[22]; therefore, it is reasonable that the PCC is involved in self-evaluation of the level of physical fatigue. However, it is unclear whether the PCC is involved in self-evaluation of other types of fatigue, such as mental fatigue.

Mental fatigue, defined as a state of reduced mental alertness that impairs performance [23], is prevalent in modern society [24] and is one of the most significant causes of accidents [25], [26]. Thus, it is essential to clarify the neural mechanisms of mental fatigue as well as those of physical fatigue. Therefore, our aim of this study was to investigate the neural mechanisms underlying self-evaluation of the level of mental fatigue. Since the neural mechanisms of self-evaluation of the level of mental fatigue may involve several brain regions, we used MEG with high temporal resolution to assess the neural activities caused by evaluating the level of mental fatigue. We performed the ECD analyses of the MEG data to enable comparison with the results of our previous study. In addition, we performed spatial filtering analyses of the MEG data to detect neural activities which were undetectable using the ECD analyses.

## Materials and Methods

### Participants

Fourteen healthy male volunteers (22.9±3.6 years of age [mean ± SD]) participated in this study. All participants were right-handed according to the Edinburgh Handedness Inventory [27]. Current smokers, individuals with a history of mental or brain disorder, and individuals taking chronic medications that affect the central nervous system were excluded. The Ethics Committee of Osaka City University approved the study protocol. All participants provided written informed consent for participation in this study in accordance with the principles of the Declaration of Helsinki.

### Experimental Design

**Figure 1:**
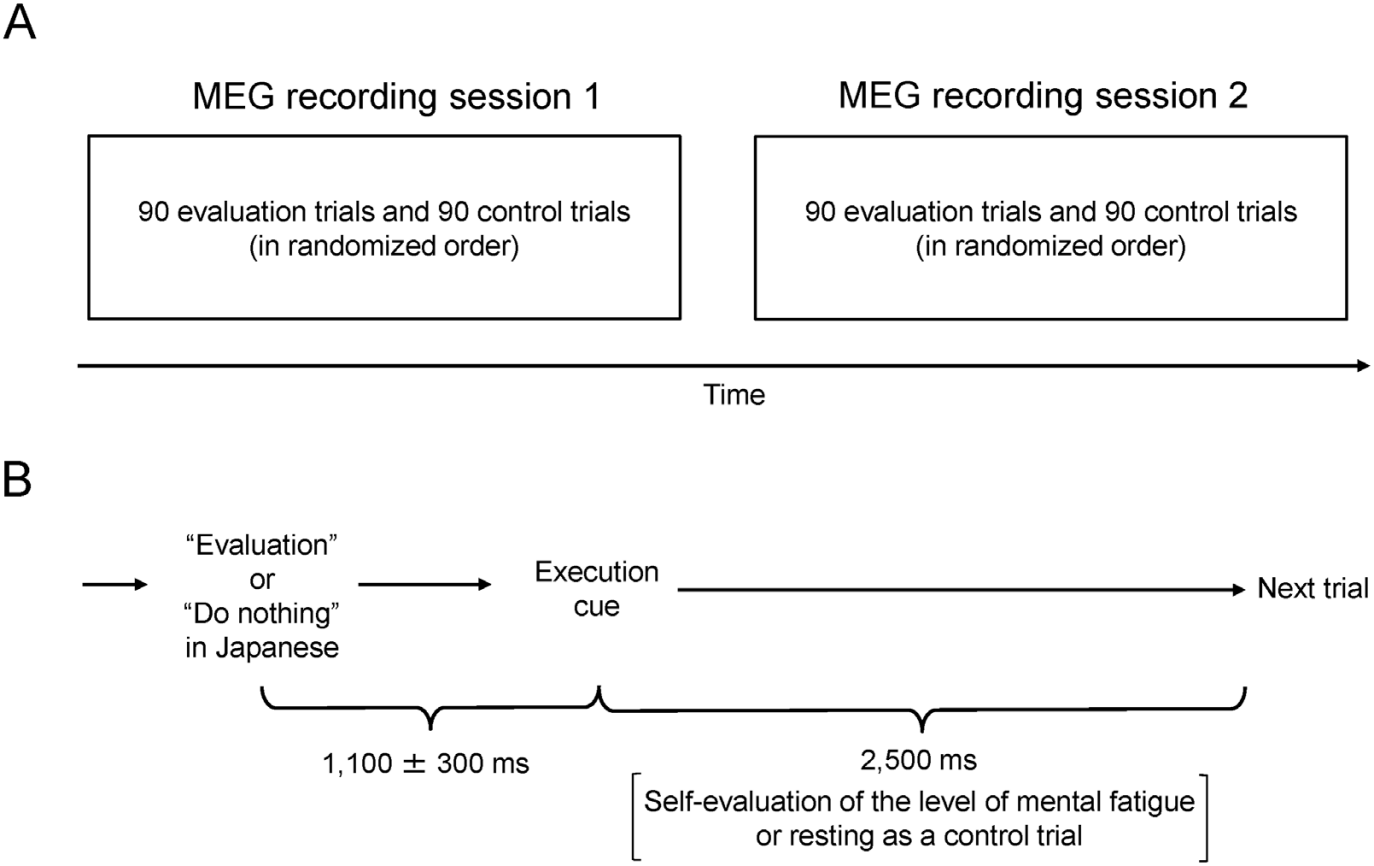
Experimental design. This experiment consisted of two identical magnetoencephalography (MEG) recording sessions. Each session was composed of 90 evaluation trials and 90 control trials and the order of the trial presentation was randomized (A). In the evaluation trials participants were asked to evaluate the level of mental fatigue in time with execution cue that followed an announcement that the upcoming trial is an evaluation trial. In the control trials, participants were asked to do nothing in time with the execution cue that followed an announcement that the upcoming trial is a control trial. The interval between the announcement and the execution cue was 1,100±300 ms (mean ± SD), where the jitter was generated based on Gaussian distribution. The next trial started 2,500 ms after the onset of the execution cue (B).

The experiment consisted of two identical MEG recording sessions. Each session was composed of 90 evaluation trials and 90 control trials and the order of the trial presentation was randomized (Figure 1A). The participants lay on a bed in a magnetically shielded room in the supine position with their eyes closed and were asked to listen to cue sounds (440-Hz sine wave tone with a duration of 30 ms) played on a personal computer using OpenSesame software [28]. In evaluation trials participants were asked to evaluate their level of mental fatigue. An announcement that the upcoming trial is an evaluation trial was made in Japanese, and the participants were instructed to evaluate their level of mental fatigue when they heard an execution cue. The interval between the announcement and the execution cue was 1,100±300 ms (mean ± SD), with the jitter generated based on Gaussian distribution. The next trial started 2,500 ms after the onset of the execution cue. In control trials participants received an announcement that the upcoming trial is a control trial and were not required to take any action on the subsequent execution cue. The interval between the announcement and the execution cue and between the execution cue and the start of the next trial were the same as in the evaluation trials (Figure 1B). To assess the relation between neural activity and the daily level of fatigue sensation, all participants completed the Japanese version of the Checklist Individual Strength (CIS) questionnaire [29], [30], which consists of 20 questions and is designed to assess the daily level of fatigue sensation. To ensure that the participants successfully evaluate the level of fatigue in the evaluation trials and not to evaluate the level of fatigue in the control trials, they performed the evaluation and control trials several times prior to the first MEG session until they felt that they could perform properly in the evaluation and control trials.

### MEG Recordings

MEG recordings were performed using a 160-channel whole-head type MEG system (MEG vision; Yokogawa Electric Corporation, Tokyo, Japan) with a magnetic field resolution of 4 fT/Hz^1/2^ in the white-noise region. The sensor and reference coils were gradiometers with 15.5-mm diameter and 50-mm baseline, and the two coils were separated by 23 mm. The sampling rate was 1,000 Hz with a 0.3 Hz high-pass filter.

### MEG Analyses

In our previous study we analyzed MEG data using an ECD method [15]. ECD methods are based on the assumption that the number of neural activities observed at a given time is limited [31], [32], and it is difficult to reliably detect neural activities using ECD methods when multiple brain regions are activated at one time [33], [34]. In addition, ECD methods require the temporal pattern of brain activation to be time- and phase-locked to a stimulus [35]–[37]. This means that cognitive processes that include jitter in the latency of neural activations cannot be detected using ECD methods [35]. Therefore, in addition to the ECD analyses, we analyzed the MEG data using a spatial filtering method, which can detect time-locked but phase-unlocked activities regardless of the number of neural activities or brain regions activated at one time. We used narrow-band adaptive spatial filtering as the spatial filtering method [32], [38] and assessed the dynamics of oscillatory brain activities, which reflect time-locked cortical activities [35], [36], [39].

The MEG data obtained in the MEG recording session 1 and 2 were combined into one MEG file by participants, including 180 evaluation and 180 control trials. Before processing the MEG data the magnetic noise that originated from outside the magnetically shielded room was eliminated by subtracting the data obtained from reference coils using specialized software (MEG 160; Yokogawa Electric Corporation). ECD analysis was performed using MEG 160 software to identify the activities evoked by evaluation of the level of mental fatigue. MEG data corresponding to evaluation and control trials were separately averaged offline after analogue-to-digital conversion and band-pass filtered at 3–30 Hz [14], [15], [40]–[42]. In each channel, the mean signal in the pre-stimulus baseline period (from 0 to 500 ms before the start of the execution cue) was subtracted from the data to remove any baseline shift. Epochs of the raw MEG data that included artifacts were identified by careful visual detection and were excluded from the analyses before averaging. The following parameters were estimated from ECD analyses: latencies, intensities, and three-dimensional locations and orientations based on individual magnetic resonance imaging (MRI) coordinates. The dipole assignment required a goodness of fit (GOF) value above 85%, i.e., the ECD must explain >85% of the field variance, based on a previous report [43]. We estimated ECDs with GOF value more than 85% as many as possible across time range from 0 to 500 ms based on the sink and source patterns in the isofield contour maps and magnetic waveforms in the evaluation and control sessions for each participant. Since neural activity in the PCC has been reported to be related to the self-evaluation of the level of physical fatigue [15], our aim was to examine whether ECDs can also be estimated in the PCC when the participants evaluate their level of mental fatigue.

In addition to the ECD analysis, spatial filtering analysis of the MEG data was performed to identify activity that was caused by evaluation of the level of mental fatigue. The MEG data were band pass filtered at 1–4 Hz, 4–8 Hz, 8–13 Hz, 13–25 Hz and 25–58 Hz by a finite impulse response filtering method using Brain Rhythmic Analysis for MEG software (BRAM; Yokogawa Electric Corporation) to obtain delta, theta, alpha, beta and gamma signals, respectively. After the band pass filtering, the location and intensity of cortical activity was estimated using BRAM, which uses a narrow-band adaptive spatial filtering algorithm [32], [38]. Voxel size was set at 5.0×5.0×5.0 mm. Oscillatory power ratios for the evaluation and control trials were separately calculated. Oscillatory power ratio for each frequency band was calculated relative to baseline for 100-ms time windows from 0 to 1000 ms after the onset of the execution cue. Epochs that included artifacts were excluded from the analyses as in the ECD analysis. These data were then analyzed using statistical parametric mapping (SPM8, Wellcome Department of Cognitive Neurology, London, UK), implemented in Matlab (Mathworks, Sherbon, MA). The MEG parameters were transformed into the Montreal Neurological Institute template of T1-weighed images [44] and applied to the MEG data. The anatomically normalized MEG data were filtered with a Gaussian kernel of 20 mm (full-width at half-maximum) in the x-, y-, and z-axes. To enable inferences to be made at a population level, individual data were summarized and incorporated into a random-effect model [45]. The weighted sum of the parameters estimated in the individual analysis consisted of “contrast” images, which were used for the group analyses [45]. The resulting set of voxel values for each comparison constituted an SPM of the t statistic (SPM{*t*}). The SPM{*t*} was transformed to the units of normal distribution (SPM{Z}). Significant signal changes from the control to the evaluation trials for each contrast were assessed using *t* statistic on a voxel-by-voxel basis [45]. The threshold for the SPM{*t*} of group analyses was set at *P*<0.05 (corrected for multiple comparisons).

### MRI Overlay

Anatomical MRI was performed using a Philips Achieva 3.0 TX (Royal Philips Electronics, Eindhoven, the Netherlands) to permit registration of magnetic source locations with their respective anatomical locations. Before MRI scanning, five adhesive markers (Medtronic Surgical Navigation Technologies Inc., Broomfield, CO) were attached to the skin of the head: Two markers at 10 mm in front of the left tragus and right tragus, one marker at 35 mm above the nasion, and two markers at 40 mm either side of the marker above the nasion. The MEG data were superimposed on MR images using information obtained from these markers and MEG localization coils.

### Statistical Analyses

Values are presented as mean ± SD unless otherwise stated. The relation between the change in power intensity for each frequency band from control to evaluation trials in Brodmann’s area (BA) 31 and BA 9 and the relation between the change in power intensity for each frequency band from control to evaluation trials and the CIS score were evaluated using Pearson’s correlation analyses. The proportion of participants in whom an ECD was estimated in the PCC was compared between control and evaluation trials using a McNemar test. The McNemar test is a statistical method used to determine whether the row and column marginal frequencies in a 2×2 contingency table are equal or unequal [46]. All *P* values were two-tailed, and values less than 0.05 were considered statistically significant. Statistical analyses were performed using IBM SPSS 21.0 software package (IBM, Armonk, NY).

## Results

### CIS Scores

The total CIS score was 55.5±13.5. The CIS has four subscales: subjective feeling of fatigue, concentration, motivation, and physical activity. The scores for each of the four were 19.3±8.4, 15.8±5.2, 9.9±4.2, and 10.6±3.1, respectively.

### ECD Analysis of MEG Data

**Figure 2:**
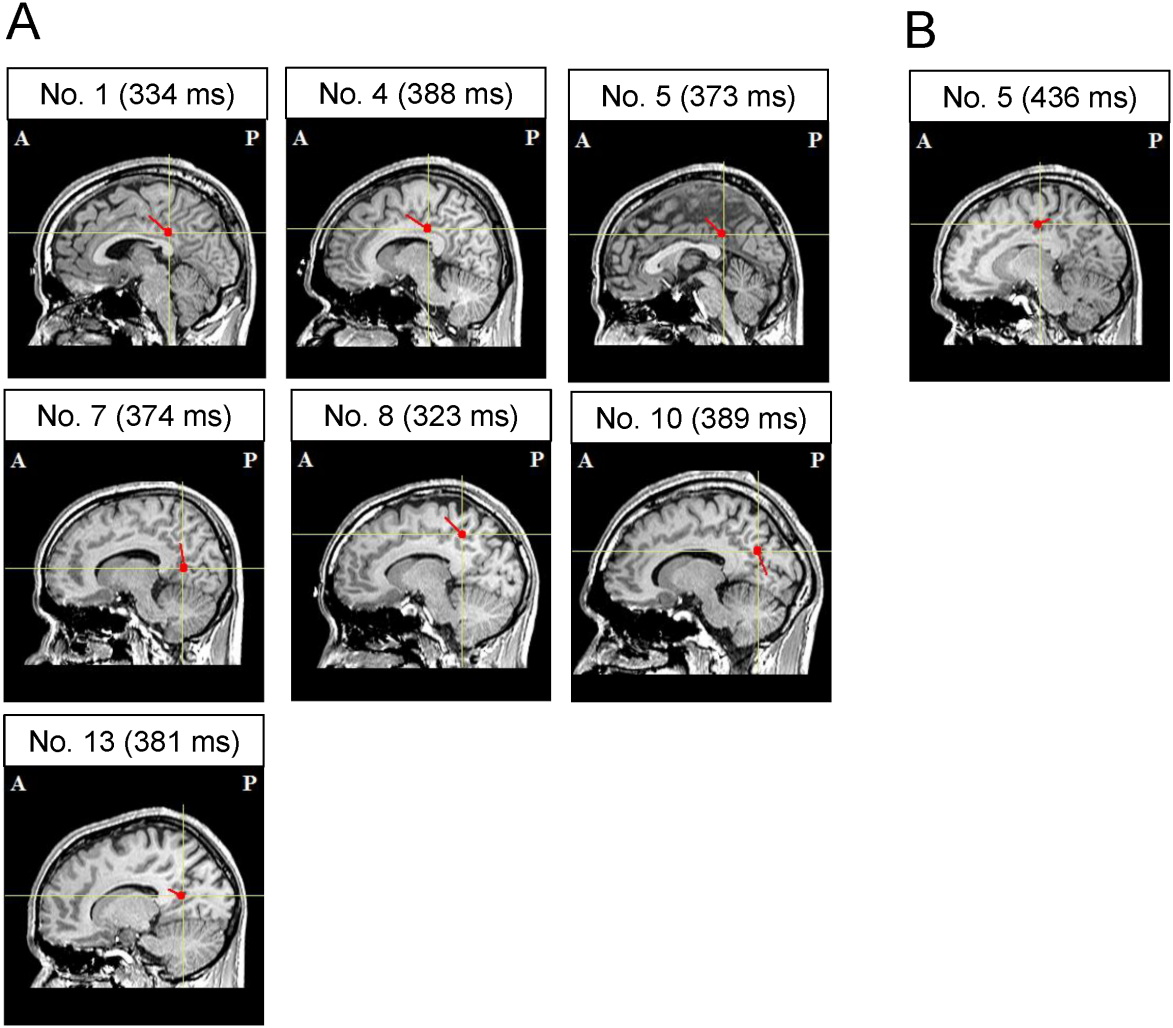
Typical magnetic waveform (A) and associated isofield contour map (B) observed during evaluation of the level of mental fatigue. The magnetic response was observed only in evaluation trials. The peak latency was 388.0Figure 2 is participant No. 4 in Figure 3. Blue solid line indicates the time point of 0 ms. Red solid line indicates the time point of 388 ms. R, right; L, left.

**Figure 3:**
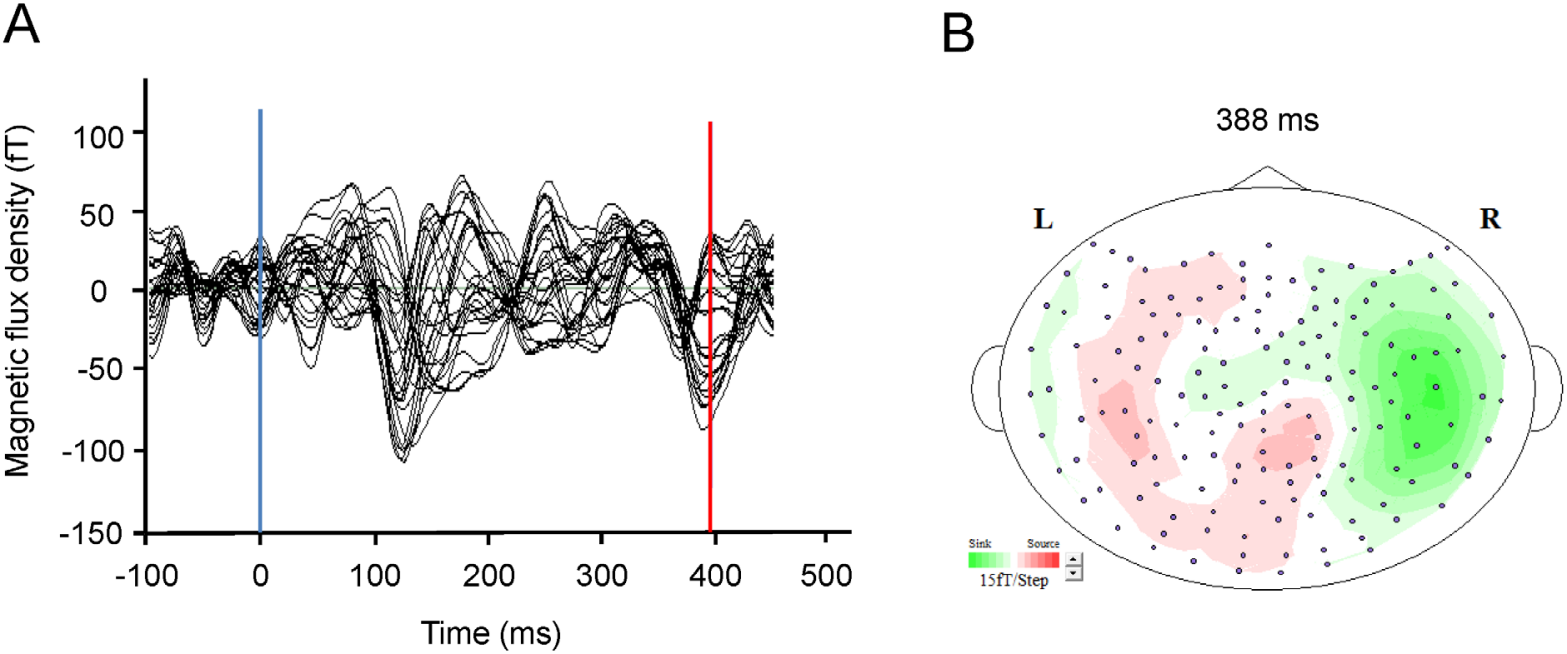
Locations of the equivalent current dipoles (ECDs) observed in evaluation (A) and control (B) trials. In evaluation trials, magnetic responses with a mean latency of 366.0(PCC) in seven of 14 participants (sagittal view shown). In control trials, a magnetic response with a latency of 436 ms was localized in the PCC in one of 14 participants (sagittal view shown). Closed red circles indicate the location of the ECD and short red lines radiating from the closed circles indicate the orientation of the ECD. ECDs are superimposed on individual MRIs. The peak latencies are indicated in parentheses. A, anterior; P, posterior.

There were several magnetic responses both in the evaluation and control trials including M100 responses produced by auditory stimuli. All the estimated ECDs were located in the PCC, except for these auditory responses to the execution cue observed within the time range of 0–200 ms. Thus, we examined whether ECDs can be estimated in the PCC when the participants evaluate their level of mental fatigue. In the evaluation trials, we observed ECDs in the PCC in seven of 14 participants, with a mean latency of 366.0 ms (Figure 2A). In the control trials, we observed ECDs in the PCC in only one of 14 participants, and the latency was 436.0 ms (Figure 2B). A typical example of the magnetic waveform in which the ECD in the PCC was estimated in the evaluation trials is shown in Figure 3A (participant No. 4 in Figure 2A). The isofield contour map corresponding to this ECD is shown in Figure 3B. In this participant, no ECDs in the PCC with GOF value more than 85% were estimated in the control trials across the time range from 0 to 500 ms. The proportion of the participants in whom ECDs could be estimated in the PCC was significantly higher in the evaluation trials than in the control trials (*P*<0.05, McNemar test). The laterality of the ECDs observed in the PCC in the evaluation trials was left in all participants. The mean latency, GOF value, and intensity of ECDs are summarized in Table 1.

**Table 1:** Properties of the equivalent current dipoles observed in in the posterior cingulate cortex during evaluation and control trials.

	Evaluation trials	Control trials
N (%) of participants	7 (50%)	1 (7%)
Peak latency (ms)	366.0±26.5	436
GOF (%)	93.9±3.4	90.9
Intensity (nAm)	21.6±8.1	18.7

Data are n (%), mean, or mean ± SD. GOF, goodness of fit.

### Spatial Filtering Analysis of MEG Data

**Figure 4:**
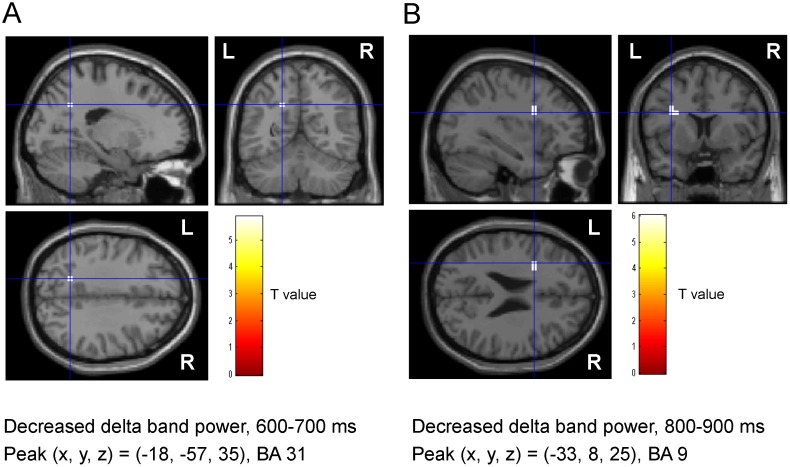
Statistical parametric maps of decreased delta (1–4 Hz) band power in the time windows of 600–700 ms (A) and 800–900 ms (B) after the onset of the execution cue in the evaluation trials relative to the control trials (paired t-test). Statistical parametric maps are superimposed on surface-rendered high-resolution MRI. The extent of the decreased delta band power 600–700 ms after the onset of the execution cue in the brain region extending from Brodmann’s area (BA) 31 (A) and delta band power 800–900 ms after the onset of the execution cue in the brain region extending from BA 9 (B) were greater in evaluation trials than in control trials. The right (R) and left (L) sides are indicated. Random-effect analyses of 13 participants, *P*<0.05, corrected for the entire search volumes (family-wise error rate).

**Figure 5:**
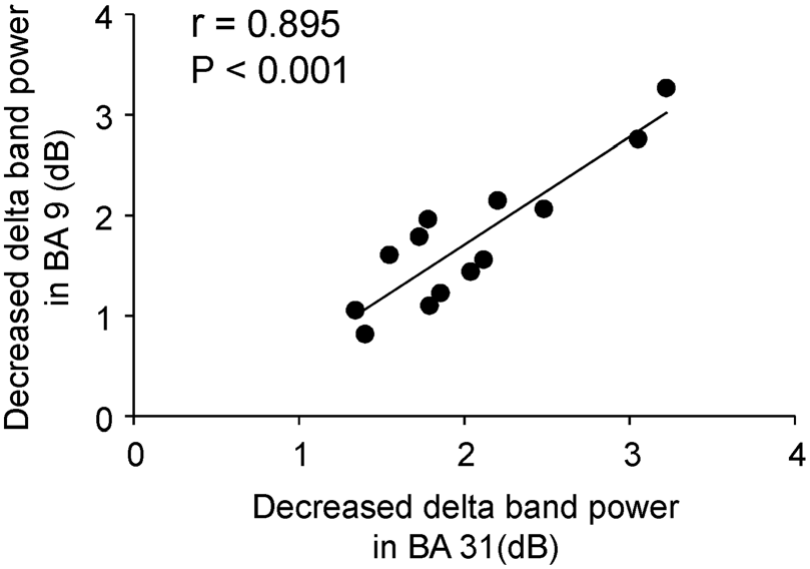
Relation between the decrease in delta band (1–4 Hz) power in evaluation trials relative to control trials in Brodmann’s area (BA) 31 and BA 9. The peak delta band power in BA 31 and BA 9, which are shown in Figure 4, were used. The linear regression line, Pearson’s correlation coefficient, and *P* value are shown.

**Figure 6:**
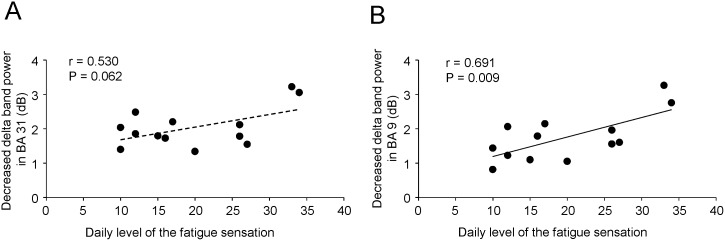
Relations between the decrease in delta band (1–4 Hz) power in evaluation trials relative to control trials in Brodmann’s area (BA) 31 (A) and BA 9 (B) and the daily level of fatigue sensation. Linear regression lines, Pearson’s correlation coefficients, and *P* values are shown.

MEG data from one participant (participant No. 6) were excluded from the spatial filtering analyses because the software did not accept the data for an unknown reason. To identify the brain regions associated with the self-evaluation of mental fatigue, power in delta, theta, alpha, beta, and gamma frequency bands was calculated relative to baseline in 100-ms windows from 0 to 1000 ms after the onset of the execution cue, and compared between the evaluation trials and the control trials. Decreased delta band power was observed in the PCC (BA 33) in the time window of 600–700 ms (Figure 4A; *P*<0.05, corrected for the entire search volumes using family-wise error rate) and the extent of the decrease was greater in evaluation trials than in control trials. Decreased delta band power was also observed in the left dorsolateral prefrontal cortex (DLPFC; BA 9) in the time window of 800–900 ms and the extent of the decrease was greater in evaluation trials than in control trials (Figure 4B; *P*<0.05). There were no significant power changes in theta, alpha, beta or gamma frequency bands. The decrease in delta band power in the PCC in the time window of 600–700 ms was positively correlated with the decrease in delta band power in the DLPFC in the time window of 800–900 ms (r = 0.895, *P*<0.001; Figure 5). The decrease in delta band power in the DLPFC in the time window of 800–900 ms was positively correlated with the daily level of fatigue assessed using the CIS (r = 0.691, *P* = 0.009; Figure 6B).

## Discussion

In the present study, we identified brain regions activated by self-evaluation of the level of mental fatigue by analyzing MEG data using ECD and spatial filtering methods. ECDs in the PCC were observed in seven of 14 participants when evaluating the level of mental fatigue. The extent of the decreased delta band power in the PCC (BA 31) was greater in the evaluation trials than the control trials in the time window of 600–700 ms after the onset of the execution cue, and this was followed by decreased delta band power in the DLPFC (BA 9) in the time window of 800–900 ms. The decrease in delta band power in the DLPFC was positively associated with that in the PCC and positively associated with the daily level of fatigue sensation.

ECDs in the PCC observed when participants evaluated the level of mental fatigue had a latency of 366.0±26.5 ms. In our previous study we reported that ECDs in the PCC observed when participants evaluated the level of physical fatigue had a latency of 378.8±18.1 ms [15]. This indicates that evaluation of physical and mental fatigue share common neural substrates, i.e., the PCC. However, in contrast with our previous study in which ECDs in the PCC were observed in most (nine of 10) participants, ECDs in the PCC were observed in only seven of 14 participants in our present study. In our present study, it was difficult to estimate ECDs in some participants because the contour map of the MEG data showed multiple sources and sinks, i.e., multiple regions of brain were simultaneously activated in the evaluation trials. This suggests that the neural mechanisms underlying the evaluation of mental fatigue are more complex than those of physical fatigue. Therefore, we performed spatial filtering analyses of the MEG data following the ECD analyses.

The neural activity in the PCC was also observed when MEG data were analyzed using the spatial filtering method. The extent of the decreased delta band power in the PCC 600–700 ms after the onset of the execution cue was greater in the evaluation trials than in the control trials, and this decreased delta band power in the PCC was followed by decreased delta band power in the DLPFC at 800–900 ms after the onset of the execution cue. Oscillatory delta activity is involved in cognitive processes, including processing of emotional [47], [48] and painful stimuli [49], and we interpret these data as showing that the PCC and DLPFC are involved in the evaluation of mental fatigue. The positive association between the decrease in delta band power in the DLPFC and the PCC indicates functional association between the DLPFC and PCC. It has been reported that the PCC is involved in self-evaluation of various sensations such as pain [16]–[18], itch [19], heating stimulation [20], and thirst [21], and it has also been suggested that the PCC is involved in the fatigue sensation [11], [12], [14]. Together with the findings of our present and previous [15] studies, these data indicate that the PCC may be involved in self-evaluation of fatigue.

The DLPFC has been reported to be involved in the neural mechanisms that determine performance under conditions of fatigue [50], [51]. Either a neural mechanism that enhances work output (i.e., facilitation system of fatigue) or a neural mechanism that limits work output (i.e., inhibition system of fatigue) is adopted during fatigue, and the DLPFC is involved in the selection of facilitation or inhibition according to the context of the situation. Therefore, it is plausible that the neural mechanisms underlying the evaluation of fatigue include the following steps: (1) the level of fatigue is evaluated in the PCC; (2) information regarding the level of fatigue is transferred from the PCC to the DLPFC; and (3) the DLPFC determines work output based on the information relayed from the PCC. The positive correlation between the decrease in delta band power in the DLPFC and the daily level of fatigue sensation may indicate that activity of the DLPFC is enhanced in participants with a high daily level of fatigue sensation.

We observed two aspects of neural activity in the PCC related to the evaluation of mental fatigue, i.e., ECDs with a latency of 366.0±26.5 ms and decreased delta band power in the time window of 600–700 ms after the onset of the execution cue. These two aspects of neural activity have different latencies; therefore, the PCC may be involved in several aspects of the self-evaluation of mental fatigue, including self-evaluation related to episodic memory and visuospatial orientation [52], [53]. Further study is needed to clarify this point.

Since it is essential to accurately evaluate the level of fatigue so that the fatigue sensation matches the level of fatigue in order to avoid fatigue-related problems such as overwork and chronic fatigue, the neural mechanisms of the self-evaluation of the level of fatigue seem to be related to those of fatigue sensation. There have been several studies that investigated the neural mechanisms of fatigue sensation. The regional blood flow in the medial orbitofrontal cortex assessed by using H_2_^15^O positron emission tomography was positively related to the level of fatigue sensation induced by cognitive test trials [13] and a functional magnetic resonance imaging study showed activations in the PCC and several other brain regions, which were related to the level of the fatigue sensation, induced by attention test trials [11]. Another fMRI study showed activation in the PCC when participants imagined that they were fatigued [12]. It has been reported that MEG activations were observed in the PCC when participants viewed pictures of people with fatigued expressions, suggesting involvement of the PCC in the neural mechanisms of fatigue sensation [14]. In this study, MEG activations in the insular cortex (IC) were also observed in some participants. Although these are the candidate brain regions related to the neural mechanisms of fatigue sensation, the role of each brain region in the neural mechanisms of fatigue sensation was unclear: The medial orbitofrontal cortex may be involved in controlling work output [13], [54] and the PCC and IC may be involved in the self-evaluation of the level of fatigue [14], [22], [52], [53], [55]–[57]. Recently, the involvement of the insular cortex in the self-evaluation of mental effort investment [58] and the involvement of the PCC in the self-evaluation of the level of physical fatigue [15] have been reported in fMRI and MEG studies, respectively. In addition to these findings, our present study showed that the PCC is involved in the neural mechanisms of self-evaluation of the level of mental fatigue as well as those of physical fatigue and that the functional association between the PCC and DLPFC seems to play an important role in the evaluation of mental fatigue sensation.

There are limitations to our study. First, since fatigue sensation is a subjective sensation, it is difficult to objectively assess whether participants adequately evaluated the level of fatigue in the evaluation trials. Instead of assessing whether participants adequately performed the task trials, we confirmed that they felt that they could perform properly in the evaluation and control trials during the pre-training period. Second, we examined the neural activity associated with evaluating the level of mental fatigue in participants with similar levels of mental fatigue. The level of fatigue of our participants was not high, as they did not perform any fatigue-inducing task trials. Testing our results in participants with a wider range of mental fatigue levels would further our understanding of the neural mechanisms that underlie fatigue evaluation. Third, although we could determine the temporal sequence of brain activity, it is difficult to discuss the precise location of the activated brain areas because of the low spatial resolution of MEG. Additional studies with other neuroimaging techniques, such as fMRI are necessary to overcome this problem. Fourth, since there have been numerous MEG studies in which ECDs in the PCC were successfully estimated and the ECDs estimated in our study had clear sink and source patterns and good GOF values, we think that the ECDs in the PCC were reliably estimated in our study. However, we cannot completely exclude the possibility that the localization of the ECDs in the PCC was affected by superficial sources. Finally, the number of the participants was limited and all the participants were male. To generalize our results, studies with a larger number of participants including females are needed.

In conclusion, we showed that the neural activity in the PCC and DLPFC is involved in the self-evaluation of mental fatigue. Our data suggest a functional association between the PCC and DLPFC that seems to play an important role in the evaluation of fatigue sensation. Our findings may help clarify the mechanisms of fatigue sensation and may help the development of treatment methods for patients who suffer from severe fatigue sensation.
